# Analysis of Individual Social-ecological Mediators and Moderators and Their Ability to Explain Effect of a Randomized Neighborhood Walking Intervention

**DOI:** 10.1186/1479-5868-6-49

**Published:** 2009-07-30

**Authors:** Yvonne L Michael, Nichole E Carlson

**Affiliations:** 1 Department of Public Health and Preventive Medicine, Oregon Health and Science University, 3181 SW Sam Jackson Park Road, Portland, Oregon 97239-3098, USA; 2 Center for Health Research, Kaiser Permanente Northwest, 3800 N Interstate Avenue, Portland, Oregon 97227, USA; 3 Department of Biostatistics and Informatics, University of Colorado, 13001 E 17th Place, Aurora, Colorado 80045, USA

## Abstract

**Background:**

Using data from the SHAPE trial, a randomized 6-month neighborhood-based intervention designed to increase walking activity among older adults, this study identified and analyzed social-ecological factors mediating and moderating changes in walking activity.

**Methods:**

Three potential mediators (social cohesion, walking efficacy, and perception of neighborhood problems) and minutes of brisk walking were assessed at baseline, 3-months, and 6-months. One moderator, neighborhood walkability, was assessed using an administrative GIS database. The mediating effect of change in process variables on change in brisk walking was tested using a product-of-coefficients test, and we evaluated the moderating effect of neighborhood walkability on change in brisk walking by testing the significance of the interaction between walkability and intervention status.

**Results:**

Only one of the hypothesized mediators, walking efficacy, explained the intervention effect (product of the coefficients (95% CI) = 8.72 (2.53, 15.56). Contrary to hypotheses, perceived neighborhood problems appeared to suppress the intervention effects (product of the coefficients (95% CI = -2.48, -5.6, -0.22). Neighborhood walkability did not moderate the intervention effect.

**Conclusion:**

Walking efficacy may be an important mediator of lay-lead walking interventions for sedentary older adults. Social-ecologic theory-based analyses can support clinical interventions to elucidate the mediators and moderators responsible for producing intervention effects.

## Background

Health interventions have come to rely more heavily on social-ecological theory as recognition grows that a one-size-fits-all approach to community-level interventions is not optimal. In the field of health promotion, social-ecological theory focuses on identifying individual, social-environmental, and physical environmental influences on health behaviors. Social-ecologic theory provides a conceptual framework for informing the development of intervention strategies that target changes beyond the individual level, with an emphasis on environmental and policy influences [1,2]. Social-ecologic theory does not give specific guidance on which variables within each domain of influence are an appropriate focus, thus specific mediators and moderators must be identified for each behavior or health outcome of interest.

When interventions are designed with these influences in mind, researchers can more effectively study the effects of individual mediators or moderators on the intervention effect in question, and can thus improve efficacy of health interventions [3]. For example, identifying specific mediators or moderators of effects of neighborhood-based interventions to increase physical activity could help create supportive environments and refine future interventions based on theoretical models linking multiple levels of influence [4-8]. Analyses identifying which mediators and moderators produce the intervention effect are also crucial for ensuring that, when interventions are customized for different age groups, genders, racial/ethnic groups, or to any specific community, the elements of the intervention that produce its effect are not lost during customization. Knowing which mediators and moderators produce the intervention effect allows for researchers to pay particular attention to ensuring that mediators and moderators of the intervention effect remain culturally relevant to the community targeted by the intervention [8]. For example, a recent intervention of culturally specific dance among African American women identified mediating effects of social support on lifestyle physical activity. The authors conclude that including the relationship of social support for a specific behavior will be essential when developing interventions for other cultural groups [9].

Senior Health and Physical Exercise (SHAPE), a lay-led, neighborhood-based intervention designed to promote walking, incorporated strategies from social-ecological theory in its design, and has been shown to increase walking activity compared to an education control in a cluster-randomized controlled trial [10]. The main structural element of the SHAPE intervention, lay-led walking groups, grew directly out of social-ecological theory because walking groups were assumed to increase social cohesion, increase walking efficacy (participants' belief that they could walk for 30 minutes three times per week in the presence of barriers such as weather, time constraints, etcetera); and decrease perceptions of neighborhood problems, which would in turn increase walking activity. Although social-ecological theory predicts that these, or other, mediators are responsible for increases in physical activity resulting from interventions such as SHAPE, little work has been done analyzing each mediator or moderator separately or in combination to determine whether an individual mediator or moderator is contributing to the effect of the intervention and the extent to which effects of these mediators overlap.

Several physical activity-specific multi-level models have been proposed that include variables at the individual, social and policy levels influencing behavior change [11-14]. The study described here is one of the first to systematically identify and examine these potentially relevant mediators and moderators of neighborhood-based physical activity interventions [6,10,15-17]. The aim of the current study was to test the hypothesis that three proposed social-ecological mediators (social cohesion, walking efficacy, and perception of neighborhood problems), and one proposed moderator (neighborhood walkability), helped explain an increase in walking activity among older adults participating in the SHAPE intervention, compared to a control group. We proposed that lay-led neighborhood walking groups create opportunities to walk regularly in the neighborhood, meet neighbors, interact with non-walking group neighbors who are also outdoors, and learn about neighborhood resources and facilities through walking around. These activities enhance walking efficacy, build stocks of social cohesion, and reduce incorrect perceptions about neighborhood problems, that in turn leads to greater levels of neighborhood walking. Based on existing research linking neighborhood walkability and physical activity [18], we hypothesized that the intervention would be more successful, defined by a larger increase in walking behavior, in the highly walking-accessible neighborhoods.

### Background on the SHAPE Trial

Because the SHAPE trial was originally designed to facilitate an analysis such as the one we performed, it is important to briefly outline the structure of SHAPE and the nature of the data collected. All data used in our analysis came from the SHAPE trial; detailed information about SHAPE's sampling and recruitment has been previously reported [10]. Fifty-six neighborhoods in Portland, Oregon (population 73,828) were selected from a total of 93 neighborhoods demarcated by the Portland City Council. Low-income and high-minority neighborhoods in the city were over-sampled. A coin flip was used to randomly assign the neighborhoods to either a walking group intervention (N = 28) or an education-only control (N = 28). Residents of selected neighborhoods were eligible for inclusion in the trial if they were: (1) aged 65 years or older, (2) cognitively intact, (3) not active in a formal exercise program during the previous month, and (4) able to walk without an assistive device. A total of 582 participants were recruited (with about 10 subjects per neighborhood and an overall response rate of 30.5%) and their average age was 74 years.

### SHAPE Intervention

Participants residing in neighborhoods randomized to the intervention condition participated three times per week for six consecutive months in a leader-led walking group program. Participants in control condition neighborhoods received a health education and information program, mailed regularly during the 6-month intervention period. In an intention-to-treat analysis, intervention neighborhoods experienced significant improvements in physical and mental health-related quality of life and improved life satisfaction compared to control neighborhoods (p < 0.05) [10]. A significant increase was also observed in the secondary outcome of neighborhood walking activity, a measure of how frequently participants walked or strolled in the neighborhood, walked or did any other physical activity with neighbors, went to a neighborhood park for walks or other physical activities (p < 0.05) [10].

### Assessments

During an interview at baseline, trial participants provided information on walking activity, demographic characteristics, health status, social cohesion, walking efficacy, and neighborhood problems. Follow-up assessments collected similar data by mail or telephone at the mid-point (3 months) and conclusion of the intervention (6 months).

## Methods

### Measures

#### Outcome

We assessed self-reported brisk walking using questions from the Yale Physical Activity Scale (YPAS) [19], an instrument validated in older adults [20,21]. These data were gathered, but not assessed, during the SHAPE trial. For each of the three time periods, we calculated the total number of minutes of brisk walking per week by multiplying the number of times an individual engaged in brisk walking per week by the number of minutes per walking event. For this analysis, we excluded subjects if data on brisk walking were not available for them or were out of range, e.g., indicating > 400 minutes of walking per week (n = 34), or if residential addresses could not be geocoded (n = 8).

#### Mediators of outcome

We selected neighborhood social cohesion, walking efficacy, and perceptions of neighborhood problems as potential mediators based on social-ecological theory as described previously [16]. Questions relating to mediators were included in baseline and follow-up interviews as part of the SHAPE trial.

**Social cohesion **was measured using five items developed by Sampson, Raudenbush, and Earls [22]. Respondents were asked how strongly they agreed, on a scale of 1 (strongly disagree) to 5 (strongly agree), with statements such as "people around here are willing to help their neighbors," "this is a close-knit neighborhood," "people in this neighborhood can be trusted," "people in this neighborhood generally do not get along with each other," and "people in my neighborhood do not share the same values" (Chronbach's α = 0.90). High scores indicated greater perceived social cohesion among neighborhood residents.

The **walking efficacy **scale, adapted from McAuley and Mihalko [23], contains nine items scored from 1 (not confident at all) to 5 (completely confident). These items assess the participant's belief that he or she could walk for 30 minutes three times per week in the presence of barriers (e.g., weather, vacation) (Chronbach's α = 0.96). High scores indicated greater walking efficacy.

**Perceptions of neighborhood problems **were assessed through seven items adapted from Sallis and colleagues [24]. Respondents were asked how strongly they agreed, on a scale of 1 (strongly disagree) to 5 (strongly agree), with statements related to whether gangs, graffiti, violent crime, vandalism, burglary, abandoned or boarded up buildings, or alcohol or drug use were problems in their neighborhood (Chronbach's α = 0.90). High scores indicated perceptions of more severe neighborhood problems.

#### Moderators

We developed a walkability score to evaluate the extent to which neighborhood walkability moderated the effect of the intervention. Cross-sectional evidence suggests that higher levels of walking for exercise among older adults are supported when certain built environment elements exist in a neighborhood [25,26]. Therefore, we assessed built-environment characteristics identified in the planning literature as relevant to physical activity [27] by neighborhood for each participant. Participants' baseline residential addresses were geocoded in ArcGIS 9.1 (ESRI, Redlands, CA) and linked to the Regional Land Information System (RLIS), a GIS database maintained by Metro (the greater Portland area's regional planning agency) in order to calculate measures of sidewalk coverage, connectivity, public transportation access, distribution of parks/green space, and level of automobile traffic volume within quarter-mile and half-mile radii around each residential address. We obtained data regarding number and type of retail establishments within quarter-mile and half-mile buffers around each participant's residence by incorporating publicly available directory information into the GIS layers [28].

#### Walkability

To develop the overall walkability score, we performed a principal components analysis (PCA) using the quarter- and half-mile built environment measures described above. Highly skewed variables (e.g., bus line, bus stop, walking destination frequencies) were log-transformed. We reversed distance to the nearest park in order to make direction consistent with the hypothesized association, so that a higher value indicated greater walkability.

The single factor created in PCA used built-environment characteristics at two geographic levels (quarter-mile and half-mile). This factor included 14 variables and explained 40% of the variance in overall walkability. We conducted secondary analyses using the quarter- and half-mile variables separately and obtained similar results. Variables included in the summary measure of walkability are sidewalk coverage, connectivity, public transportation access, distribution of parks/green space, and level of automobile traffic volume.

#### Neighborhood Confounders

Neighborhood-level covariates were included because the intervention was neighborhood-based and thus relevant neighborhood factors could confound the results of the intervention. Neighborhood household income data were obtained from the 1996 American Community Survey and compiled by the Office of Neighborhood Involvement in Portland, Oregon [29]. Neighborhood poverty was measured as the proportion of households in the municipally-defined neighborhood with annual incomes of less than $15,000. Perceived neighborhood safety was assessed with one item from Sallis et al. [24], which asked participants to rate the extent to which they agreed with the statement: "It is safe to walk or jog alone in my neighborhood during the day." This item was rated on a five point scale from 1(strongly disagree) to 5(strongly agree) and aggregated to the neighborhood level. A higher score indicated a greater degree of perceived safety for walking.

### Data analysis

Changes in social cohesion, walking efficacy, and neighborhood problems were examined as potential mediators of the interventions effect on brisk walking. Walkability was assessed as a potential modifier of the interventions effect on brisk walking.

#### Intervention effects on outcome and potential mediators

Hierarchical linear modeling (HLM) (SAS v9.2 PROC MIXED) [30] was used to account for the cluster randomized design of the trial to initially assess whether patterns of change over time differed between the control and treatment groups in: 1) the number of minutes of brisk walking and 2) each of the potential mediators (social cohesion, walking efficacy, and neighborhood problems). For each of the four models, the baseline, 3-month, and 6-month observations were the outcome. Time was modeled as categorical, using dummy variables, to allow for a non-linear change over time. The intervention effect was assessed by including the interaction terms between intervention and time into the model. The test of the intervention effect was performed by testing whether either of the two interaction terms differed from zero. Each of the HLMs were adjusted for the following individual and neighborhood level confounders: age, gender, race, education, perceived health, and neighborhood poverty and safety.

#### Assessing mediation

The intervention effect resulted in a change in brisk walking between baseline and 3-months in the intervention group, and then remained stable between 3-months and 6-months. Thus, in the primary analysis we focused the assessment of mediation on the change in brisk walking by modeling the 6-month brisk walking as the outcome while adjusting for baseline brisk walking [31-33]. To understand the potential influence of baseline differences on the results, in a secondary analysis we reanalyzed the data using observed 6-month change in brisk walking unadjusted for the baseline value. Although this was a randomized control trial, there were significant differences in baseline measures between the control and intervention groups. There is a possibility that the observed differences in baseline characteristics imply that the control and intervention populations are actually two different populations. In this case, the analysis adjusting for baseline could introduce bias into the results of the primary analysis [31,34,35]. In general, we report the results of the primary analysis. However, we also display the results from the secondary analysis to allow readers to evaluate differences in the findings using these two methods. These analytic issues are described further in the discussion.

To assess mediating effects, a product-of-coefficients test was used [36,37]. This test is the product of regression coefficients derived the following way: (1) estimate the effect of the intervention on changes in the potential mediators (action theory tests; α coefficient) by regressing 6-month mediator value on the intervention, after adjustment for baseline mediator value and adjusting for the confounders listed using an HLM to control for the cluster-randomized study design; (2) estimate the independent effect of change in the mediator between 6-months and baseline by regressing 6-months brisk walking values on 6-month mediator values, adjusted for intervention, baseline brisk walking, baseline mediator, and the potential confounders listed above using an HLM (conceptual theory test; β coefficient); (3) estimate the mediating effect by calculating the product of α and β and divide by the standard error of the product. The significance of the product in (3) was determined using PRODCLIN [37,38]. This process was followed for each of the three potential mediators to assess single mediator effects. To assess multiple mediator effects, the coefficients in (2) were also obtained by fitting an HLM with all three mediators simultaneously included, and the products and tests recalculated using PRODCLIN.

To investigate other potential temporal orderings of the mediating effect on the outcome, the above analyses were repeated with change in social cohesion, self-efficacy, and perceived neighborhood problems at 3-months. The outcome of interest remained brisk walking at 6-months.

In secondary analyses, all of the analyses were repeated with change in baseline brisk walking at 6-month as the outcome. Action effects were estimated by regressing change in the mediator between 6-month and baseline on intervention. The conceptual theory estimates were derived from models unadjusted for baseline brisk walking or baseline mediator value.

#### Assessing moderation

The moderating effect of walkability was assessed by including a product term of walkability and intervention into a model with brisk walking as the outcome. The model was adjusted for baseline brisk walking and the confounders considered for the mediation analysis.

## Results

Subjects in intervention and control neighborhoods were similar except in respect to gender and perceptions of neighborhood safety (see Table 1). Participants in the intervention neighborhoods were more likely to be female compared to the control neighborhoods (76.5% v. 64.9%, p < 0.05) and also reported perceptions of worse neighborhood safety on a 5-point scale (4.4 v. 4.6, p < 0.05). Walkability did not differ significantly between intervention and control neighborhoods (Table 1).

**Table 1:** Baseline individual and neighborhood measures, by treatment group

	**Mean (SD)^a ^or Percent**	
	
	**Intervention Neighborhoods** **(n = 28)**	**Control Neighborhoods** **(n = 28)**
**Participants per neighborhood**	9.36 (3.0)	9.93 (1.7)
**Individual-level characteristics**		
Age	74.8 (2.3)	74.4 (2.1)
Female (%)*	76.5	64.9
Less than high school education (%)	47.9	42.8
Very good or excellent health (%)*	90.4	80.8
**Neighborhood-level characteristics**		
Neighborhood poverty(%)^b^	24	22
Neighborhood safety*	4.4 (0.30)	4.6 (0.22)
**Neighborhood Walkability**		
Walkability score	1.2 (5.8)	3.3 (6.7)

* P < 0.05

^a ^SD = standard deviation

^b ^Percent of households in the neighborhood with incomes < $15,000.

Walkability score includes the following quarter and half-mile built environment measures: High volume streets (%), sidewalk coverage (%), intersection frequency, bus line frequency, bus stop frequency, and walking destination frequency.

### Intervention effect

The number of minutes of brisk walking differed between the control and intervention neighborhoods (p < 0.0001). Among participants living in control neighborhoods, the number of minutes of brisk walking per week did not change throughout the 6-month intervention, while those living in intervention neighborhood increased their number of minutes of brisk walking per week an average of 41 minutes per week at 3-months and maintained that increase at the 6-month period (Table 2). After adjusting for baseline differences in the number of minutes of brisk walking at baseline, subjects in the intervention neighborhoods increased their brisk walking an average of 26 (95% CI: 12, 40; Table 3 Model 1) minutes more per week than those in the control neighborhoods at 6 months (p < 0.0001).

**Table 2:** Brisk walking and potential mediators by treatment group at baseline, 3 months, and 6 months

	**Baseline**		**3 months**		**6 months**		**p-value^a^**	
				
	**N**	**Mean (SD)**	**N**	**Mean (SD)**	**N**	**Mean (SD)**	**Time**	**Intervention^x^time**
**Minutes of brisk walking**								
Control	277	58.3 (78.1)	270	60.1 (78.4)	265	58.3 (78.8)	0.86	<0.0001
Intervention	260	42.9 (68.1)	172	83.1 (66.7)	159	75.5 (69.1)	<0.0001	
**Social cohesion**								
Control	277	3.3 (0.5)	270	3.5 (0.5)	266	3.5 (0.5)	<0.0001	0.012
Intervention	260	3.2 (0.5)	170	3.5 (0.4)	158	3.5 (0.4)	<0.0001	
**Walking efficacy**								
Control	278	7.2 (2.3)	270	6.8 (2.2)	265	6.6 (2.1)	<0.0001	0.32
Intervention	262	8.0 (1.4)	172	7.8 (1.6)	160	7.7 (1.4)	0.0042	
**Perception of neighborhood problems**								
Control	277	14.5 (6.2)	269	14.5 (5.9)	266	14.1 (6.0)	0.27	0.002
Intervention	261	14.3 (6.4)	171	15.4 (5.6)	158	15.9 (5.4)	0.0032	

^a ^Adjusted for: *Individual-level Covariates*: Age, Gender, Race/ethnicity (White and Non-White), Years of education (0–12 years, ≥ 13 years), Annual household income (<$15,000, $15,000–$29,999, ≥ 30,000), General health (poor to fair, good to excellent), walking efficacy. *Neighborhood-level Covariates*: Neighborhood poverty (proportion of households in the neighborhood with incomes < $15,000), Perceived neighborhood safety ("It is safe to walk or jog alone in my neighborhood during the day," rated on a five point scale from 1 [strongly disagree] to 5 [strongly agree]).

**Table 3:** Intervention, action theory, conceptual theory, and mediating effects on change in walking behavior^a^

		**Model 1^b^**: 6 mo follow-up adjusted for baseline	**Model 2^c^**: 6 mo follow-up adjusted for baseline, mediators at 3 mo	**Model 3^d^**: 6 mo change from baseline	**Model 4^e^**: 6 mo change from baseline, mediators at 3 mo
**Physical activity outcome**		b (95% CI)	b (95% CI)	b (95% CI)	b (95% CI)
Number of minutes of brisk walking		25.64 (11.73,39.55)***	25.64 (11.73,39.55)***	37.48 (21.82,53.14)***	37.48 (21.82,53.14)***
					
**Action theory tests** **(single mediator models)**		α (95% CI)	α (95% CI)	α (95% CI)	α (95% CI)
Social cohesion		0.097 (-0.010,0.21)	0.059 (-0.032,0.15)	0.17 (0.044,0.30)*	0.13 (0.0094,0.24)*
Walking efficacy		0.50 (0.14,0.86)**	0.58 (0.27,0.86)***	0.19 (-0.19,0.56)	0.32 (-0.0011,0.65)
Perceived neighborhood problems		1.62 (0.65,2.59)**	0.86 (-0.27,1.76)	1.65 (0.56,2.75)**	0.95 (-0.076,1.97)
					
**Conceptual theory tests** **(single mediator models)**		β (95% CI)	β (95% CI)	β (95% CI)	β (95% CI)
Social cohesion		-2.12 (-16.67,12.42)	-8.73 (-23.41,5.94)	-5.69 (-19.27,7.90)	-5.97 (-19.66,7.71)
Walking efficacy		17.43 (13.21, 21.66)***	10.97 (6.43,15.51)***	12.11 (7.72,16.51)***	6.18 (1.56,10.81)**
Perceived neighborhood problems		-1.53 (-2.96,-0.11)*	-0.25 (-1.71,1.20)	-1.34 (-2.69,0.014)	-0.31 (-1.74,1.11)
					
**Mediating effects** **(single mediator models)**		αβ (95% CI)	αβ (95% CI)	αβ (95% CI)	αβ (95% CI)
Social cohesion		-0.21 (-1.84,1.23)	-0.52 (-1.98,0.38)	-0.97 (-3.73,1.25)	-0.78 (-3.01,0.91)
Walking efficacy		8.72 (2.53,15.56)**	6.36 (2.69,10.97)***	2.30 (-2.13,7.14)	1.98 (-0.014,4.95)
Perceived neighborhood problems		-2.48 (-5.60,-0.22)*	-0.22 (-1.66,1.05)	-2.21 (-5.31,-0.047)*	0.69 (-0.58,2.58)
					
**Mediating effects** **(multiple mediator model)**		αβ (95% CI)	αβ (95% CI)	αβ (95% CI)	αβ (95% CI)
Social cohesion		-0.67 (-2.62,0.65)	-0.78 (-2.60,0.35)	-1.19 (-4.00,0.94)	-0.92 (-3.23,0.74)
Walking efficacy		8.24 (2.39,14.80)**	6.61 (2.82,11.36)**	2.08 (-1.93,6.51)	2.04 (-0.013,5.09)
Perceived neighborhood problems		-1.83 (-4.68,0.32)	-0.15 (-1.56,1.14)	-1.32 (-4.03,0.77)	0.035 (-1.44,1.54)

Notes: b-regression coefficient; 95% CI-95% confidence interval; α-estimate of regression coefficient of intervention effect on 6-month walking; β-estimate of regression coefficient of mediator effect on 6-month walking. αβ-product-of-coefficient estimates.

^a ^All models are adjusted for: *Individual-level Covariates*: Age, Gender, Race/ethnicity (White and Non-White), Years of education (0–12 years, ≥ 13 years), Annual household income (< $15,000, $15,000–$29,999, ≥ 30,000), General health (poor to fair, good to excellent). *Neighborhood-level Covariate*: Perceived neighborhood safety ("It is safe to walk or jog alone in my neighborhood during the day," rated on a five point scale from 1 [strongly disagree] to 5 [strongly agree]).

^b^In the model 1 framework, the outcome for the conceptual model is the number of minutes walked at the 6 month follow-up period and the model is adjusted for the number of minutes walked at baseline. The outcomes for the action theory tests are the observed values of each potential mediator at the 6 month follow-up period and each model is adjusted for the appropriate observed value of the mediator at baseline.

^c ^In the model 2 framework, the outcome for the conceptual model is the number of minutes walked at the 6 month follow-up period and the model is adjusted for the number of minutes walked at baseline. The outcomes for the action theory tests are the observed values of each potential mediator at the 3 month follow-up period and each model is adjusted for the appropriate observed value of the mediator at baseline.

^d ^In the model 3 framework, the outcome for the conceptual model is the change in the number of minutes walked between 6 month follow-up and baseline; no adjustment for baseline is made. The outcomes for the action theory test are the difference in the 6 month and baseline observations for each of the potential mediators; no adjustment for baseline is made.

^e ^In the model 4 framework, the outcome for the conceptual model is the change in the number of minutes walked between 6 month follow-up and baseline; no adjustment for baseline is made. The outcomes for the action theory test are the difference in the 3 month and baseline observations for each of the potential mediators; no adjustment for baseline is made.

*** p < 0.001

** p < 0.01

* p < 0.05

### Action theory tests single-mediator models (intervention effect on mediators)

In both groups, social cohesion increased between baseline and 3 months and was stable between 3 and 6 months. The increase in social cohesion was larger among the intervention group compared to the control group; this intervention effect remained significant after adjustment for individual and neighborhood-level characteristics (p = 0.012, Table 2). After adjusting for baseline differences in social cohesion, social cohesion at 6 months no longer differed between the control and intervention neighborhoods (p = 0.077, Table 3 model 1) indicating that the change in social cohesion over time did not differ between the control and intervention neighborhoods. The result was unchanged in models evaluating change in social cohesion at 3 months (Table 3 model 2).

Walking efficacy decreased over time in both groups and this decrease did not significantly differ between the control and intervention neighborhoods (p = 0.38, Table 2). After adjusting for baseline differences in walking efficacy, walking efficacy at 6-months was significantly higher in the intervention neighborhoods compared to the control neighborhoods (p = 0.0072, Table 3 model 1). The result was unchanged in models evaluating change in walking efficacy at 3 months (Table 3 model 2).

Perceived neighborhood problems increased more in the intervention neighborhoods compared to the control neighborhoods (p = 0.0019, Table 2). After adjusting for baseline differences in perceived neighborhood problems, perceived neighborhood problems were higher (were perceived as worse) in the intervention neighborhoods compared to the control neighborhoods (p = 0.0012, Table 3 model 1). We observed no significant differences in change in neighborhood problems at 3 months (Table 3 model 2).

### Conceptual theory tests (associations between change in mediators and brisk walking)

Change in social cohesion at 6-months and 3-months were not associated with brisk walking at 6-months after accounting for intervention status (Table 3 models 1 and 2). A positive change in walking efficacy at 6-months 3-months were significantly associated with more brisk walking at 6-months after adjusting for intervention status (Table 3 models 1 and 2). Change in neighborhood problems at 6-months was significantly associated with brisk walking at 6-months (Table 3 model 1). Change in neighborhood problems at 3-months was not associated with brisk walking at 6-months (Table 3 model 2).

### Mediated effects single-mediator models

As expected based on the action theory and conceptual theory tests, in the single mediator model, 6-month change in social cohesion did not significantly mediate change in brisk walking (Table 3, Models 1 and 3). A positive change in walking efficacy at 6 months significantly mediated change in walking (Table 3, Models 1 and 3), while a positive change in perceived neighborhood problems at 6 months significantly suppressed the intervention effect (Table 3, Models 1 and 3). The results were similar, but attenuated when investigating change in the mediators at 3-months on walking at 6-months (Table 3, Models 2 and 4).

### Mediated effects multiple-mediator models

In the multiple mediator model, the mediation effect of change in walking efficacy remained significant, while neither change in social cohesion nor change in perceived neighborhood problems were significant mediators or suppressors.

### Moderation of walkability

Walkability did not significantly moderate the intervention effect (p = 0.97).

## Discussion

Participation in neighborhood walking groups was associated with a small-but-sustained increase in minutes engaged in brisk walking, and in level of walking efficacy over the intervention period. Contrary to our hypothesized relation between the intervention and potential mediators, the intervention was not associated with a positive change in social cohesion or in neighborhood perceptions. In fact, the intervention was associated with a significant increase in perceived neighborhood problems. In single- and multiple-mediator models, increased walking efficacy appeared to mediate the intervention effects on brisk walking at 6 months. The single-mediator results provide some evidence that perceived neighborhood problems at 6 months (but not 3 months) suppress the intervention effects on brisk walking at 6 months. The absence of a finding for perceived neighborhood problems in the multiple mediator models suggests an overlap in mediated effects of walking-efficacy and perceived neighborhood problems.

Previous analyses examining the direct effect of physical-activity interventions among adults reported mixed results [39]. In the context of this lay-led walking intervention for sedentary older adults, we hypothesized that the intervention would increase walking efficacy. Our findings, consistent with many other studies, found significant correlations between efficacy and physical-activity behavior in older adults [40-44]. This finding suggests that increasing walking efficacy is an important component of any successful future replication of the SHAPE intervention in other populations. It should be noted that in the current study, walking efficacy decreased slightly over the intervention period in the intervention group, although to a lesser degree than it decreased in the control group. The decline in the intervention group may reflect the influence of regression to the mean given that walking efficacy was fairly high (mean 8.0, SD 1.4) at baseline. After controlling for baseline level, walking efficacy was higher at 6-months in the intervention group compared to the control group.

Contrary to our hypotheses, perceptions of neighborhood problems at 6-months (but not 3-months) significantly increased in the intervention group from baseline to follow-up, relative to the control group. Although we are not aware of any previous studies assessing perceptions of neighborhood problems as a potential mediator of an activity-intervention effect, many studies have reported a significant inverse correlation between perception of neighborhood problems and physical activity or physical function in older adults [45-47]. In contrast, King and colleagues [48] reported that greater levels of neighborhood walking were correlated with higher reports of neighborhood problems, and hypothesized that regular walkers are more familiar with problems in the neighborhood and thus more likely to report these problems. Thus, our finding of increased reports of neighborhood problems within the intervention group at 6-months is consistent with King's results. The lack of a result at 3-months may suggest that it takes longer than 3-months of walking in the neighborhood to become familiar enough to register significant neighborhood problems.

Social cohesion can be defined as a "collective dimension of society external to the individual [49]," operationalized as strong social ties, mutual trust, and reciprocity. Social cohesion is associated with higher levels of self-rated health and lower morbidity and mortality [50-52]. social-ecological theory suggests that lay-led neighborhood walking groups may increase social cohesion by creating opportunities for neighbors to meet, to interact with non-walking group neighbors who are also outdoors, and to learn about neighborhood resources and facilities [53,54]. Level of social cohesion is independently associated with health outcomes among older adults [55]. However, based on the findings from this study, changes in social cohesion were not causally related to higher amounts of brisk walking.

The effects of the walking intervention were not significantly moderated by neighborhood walkability. Previous studies in older adults have reported positive associations between neighborhood built-environment characteristics and physical activity [25,26,56-58]. Our data suggest that neighborhood walkability may not be an important concern when targeting neighborhood-based walking interventions. Other studies should further assess whether this pattern holds true.

Our study is the first to evaluate change in social-ecological process variables within a multilevel RCT, and to apply a formal statistical test of mediation. This study lays out a method for future researchers seeking to identify individual mediators and moderators, and to quantify their influence on intervention effects. One reason our study was able to perform these analyses is that when the SHAPE trial was implemented, it was done so with the idea that social-ecological theory could be used subsequently to perform just such analyses. This is important, both from the point of view of initial intervention design, and because without an understanding of which elements of an intervention are producing the effect, interventions will not be able to be successfully reproduced in other communities and populations. Thus, analyses such as we present here may, when mediators and moderators are identified that do explain intervention effects, can afford enormous cost-savings for interventionists attempting to adapt existing intervention frameworks to a variety of communities.

Although SHAPE was designed to support analyses such as the ones described here, this area of research is in its infancy. Therefore, it should not be surprising or discouraging that initial attempts to identify mediators and moderators may not initially hit upon all the mediators and moderators contributing the most to the intervention effect. It is only through systematic analyses of individual mediators and moderators that we will be able to, eventually, identify the ones making the most significant contributions to intervention effects, and use this knowledge to refine future interventions built upon the platforms of existing ones.

We of course cannot entirely dismiss the idea that our analyses did not show that all the mediators and moderator in this study had no significant influence on the effect due to the study's lack of sufficient statistical power or model misspecification, or due to confounders such as reciprocal effects [36]. Reciprocal effects are a potential explanation because of the correlative nature of change in brisk walking and mediators in this study. We attempted to address the correlative nature of the data by also evaluating mediators after three months in relation to six-month change in brisk walking. However, future studies should consider whether initial changes in mediators resulted in maintenance of increased brisk walking beyond the intervention.

There is a possibility that the observed differences in baseline characteristics were not due to random chance, in which case, adjusting for baseline values could introduce regression to the mean biases [31,35]. To investigate possible the influence of baseline differences, we conducted a secondary analysis using observed changes in walking unadjusted for baseline values as the outcome. In this secondary analysis, the action theory tests indicate that there are intervention effects on change in social cohesion and no intervention effects on walking efficacy; however, the conclusions about mediating effects of social cohesion and perceived neighborhood problems remained unchanged. Of note, when using this analytical approach, walking efficacy no longer significantly mediated the intervention effect. This change should be noted when interpreting the finding regarding walking efficacy, as should the importance of obtaining balanced groups at baseline when designing future RCTs.

The analyses were performed using data gathered from RCT participants who completed the study, and therefore the results are generalizable to the subjects who completed the study. As previously reported, attrition was higher in the intervention group; only 62% of the intervention group (n = 159) completed the final 6-month assessment, compared to 95% of the control group (n = 265) [10]. The attrition in the intervention group suggests that additional strategies geared toward retaining participants would be useful in future neighborhood-based lay-led walking interventions in older adults. Since most of the drop-out occurred by 3 months, use of traditional techniques such as imputation is limited because the only data available on the subjects are baseline data. We note that those with complete follow-up data are similar to the group without complete follow-up data. The two groups did not differ in terms of gender (p = 0.55), education (p = 0.61), self-rated health (p = 0.16), baseline walking (p = 0.66) or social cohesion (p = 0.66). In addition, if we assume that those who dropped out had no change in walking behavior and reanalyze the data, the results are consistent with our primary analysis.

Finally, measurement of walking in this study was self-reported, and thus subject to misclassification. The use of instruments to objectively measure walking, such as pedometers, is desirable for future research seeking to expand on these results [59].

## Conclusion

Our analyses showed that only one of the hypothesized mediators we identified, walking efficacy, explained the intervention effect of an increase in brisk walking in the intervention group. Contrary to our hypotheses, perception of neighborhood problems may exert a suppressor effect on brisk walking in lay-led walking interventions in older adults. Social cohesion and neighborhood walkability were not significantly related to the intervention effect in this study.

Perhaps the most important aspect of this study, however, is that it shows how social- ecologic theory-based analyses can support clinical interventions, in order to elucidate which mediators and moderators are responsible for producing intervention effects. When mediators and moderators can be correctly identified that are responsible for significant intervention effects, interventions should be able to be more effectively tailored to a wider variety of communities, at lower cost, and with better results.

## Competing interests

The authors declare that they have no competing interests.

## Authors' contributions

YLM conceived the idea of this study and interpreted the results of the data analysis and wrote the original draft. NEC prepared the data and undertook data analysis and interpretation and contributed to writing this manuscript. Both authors have read and approved the final manuscript.

## References

[B1] Cohen DA, Scribner RA, Farley TA (2000). A structural model of health behavior: a pragmatic approach to explain and influence health behaviors at the population level. Prev Med.

[B2] Green LW, Richard L, Potvin L (1996). Ecological foundations of health promotion. Am J Health Promot.

[B3] Cerin E, Taylor LM, Leslie E, Owen N (2006). Small-scale randomized controlled trials need more powerful methods of mediational analysis than the Baron-Kenny method. J Clin Epidemiol.

[B4] Bauman A (2005). The physical environment and physical activity: moving from ecological associations to intervention evidence. J Epidemiol Community Health.

[B5] Humpel N, Owen N, Leslie E (2002). Environmental factors associated with adults' participation in physical activity: a review. Am J Prev Med.

[B6] King AC, Stokols D, Talen E, Brassington GS, Killingsworth R (2002). Theoretical approaches to the promotion of physical activity: forging a transdisciplinary paradigm. Am J Prev Med.

[B7] Saelens BE, Sallis JF, Frank LD (2003). Environmental correlates of walking and cycling: Findings from the Transportation, Urban Design, and Planning Literatures. Ann Behav Med.

[B8] Sallis JF, Owen N, Glanz K, Rimer BK, Lewis FM (2002). Ecological Models of Health Behavior Health Behavior and Health Education: Theory, Research, and Practice.

[B9] Murrock CJ, Madigan E (2008). Self-efficacy and social support as mediators between culturally specific dance and lifestyle physical activity. Research and theory for nursing practice: An international journal.

[B10] Fisher KJ, Li F (2004). A community-based walking trial to improve neighborhood quality of life in older adults: a multilevel analysis. Ann Behav Med.

[B11] Ball K, Bauman A, Leslie E, Owen N (2001). Perceived environmental and social influences on walking for exercise in Australian adults. Prev Med.

[B12] Booth ML, Owen N, Bauman A, Clavisi O, Leslie E (2000). Social-cognitive and perceived environment influences associated with physical activity in older Australians. Prev Med.

[B13] Booth SL, Sallis JF, Ritenbaugh C, Hill JO, Birch LL, Frank LD, Glanz K, Himmelgreen DA, Mudd M, Popkin BM, Rickard KA, St Jeor S, Hays NP (2001). Environmental and societal factors affect food choice and physical activity: rationale, influences, and leverage points. Nutr Rev.

[B14] King AC, Jeffery RW, Fridinger F, Dusenbury L, Provence S, Hedlund SA, Spangler K (1995). Environmental and policy approaches to cardiovascular disease prevention through physical activity: issues and opportunities. Health Educ Q.

[B15] Fisher KJ, Ritchie CB, Abernethy PJ, Hutchins CA, Ford C, Miller R (1998). Just walk it: Enhancing community participation in physical activity. Health Promot J Austr.

[B16] Giles-Corti B (2006). People or places: What should be the target?. J Sci Med Sport.

[B17] Lamb SE, Barlett HP, Ashley A, Bird W (2002). Can lay-led walking programms increase physical activity in middle aged adults? A randomized controlled trial. J Epidemiol Community Health.

[B18] Saelens BE, Sallis JF, Frank LD (2003). Environmental correlates of walking and cycling: findings from the transportation, urban design, and planning literatures. Ann Behav Med.

[B19] Dipietro L, Caspersen CJ, Ostfeld AM, Nadel ER (1993). A survey for assessing physical activity among older adults. Med Sci Sports Exerc.

[B20] Harada ND, Chiu V, King AC, Stewart AL (2001). An evaluation of three self-report physical activity instruments for older adults. Med Sci Sports Exerc.

[B21] Schuler PB, Richardson MT, Ochoa P, Wang MQ (2001). Accuracy and repeatability of the Yale physical activity survey in assessing physical activity of older adults. Percept Mot Skills.

[B22] Sampson RJ, Raudenbush SW, Earls F (1997). Neighborhoods and violent crime: A multilevel study of collective efficacy. Science.

[B23] McAuley E, Mihalko SL, Duda JL (1998). Measuring exercise-related self-efficacy. Advances in sport and exercise physiology measurement.

[B24] Sallis JF, Johnson MF, Calfas KJ, Caparosa S, Nichols JF (1997). Assessing perceived physical environmental variables that may influence physical activity. Res Q Exerc Sport.

[B25] Berke EM, Koepsell TD, Moudon AV, Hoskins RE, Larson EB (2007). Association of the built environment with physical activity and obesity in older adults. Am J Public Health.

[B26] Nagel C, Michael YL, Carlson NE, Bosworth M (2008). The relationship between neighborhood built environment and walking activity among older adults. Am J Epidemiol.

[B27] Handy SL, Boarnet MG, Ewing R, Kinngsworth RE (2002). How the built environment affects physical activity: Views from urban planning. Am J Prev Med.

[B28] Michael YL, Green MK, Farquhar SA (2006). Neighborhood design and active aging. Health Place.

[B29] Office of Neighborhood Involvement Portland neighborhood demographic profiles from the 1996 ACS data. Available upon request from the corresponding author.

[B30] Raudenbush SW, Bryk AS (2002). Hierarchical Linear Models.

[B31] Fitzmaurice GM, Laird NM, Ware JH (2004). Applied Longitudinal Analysis.

[B32] Senn S (2006). Change from baseline and analysis of covariance revisted. Stat Med.

[B33] Twisk J, Proper (2004). Evaluation of the results of a randomized controlled trial: how to define changes between baseline and follow-up. J Clinical Epidemiol.

[B34] Boshuizen HC (2005). RE: Twisk and Proper: evaluation of the results of a randomized controlled trial: how to define changes between baseline and follow-up. J Clinical Epidemiol.

[B35] Van Breukelen GJ (2006). ANCOVA versus change from baseline had more power in randomized studies and more bias in nonrandomized studies. J Clin Epidemiol.

[B36] MacKinnon DP, Cazares A, Beatty LA (1994). Analysis of mediating variables in prevention and intervention research. Scientific methods for prevention intervention research NIDA Research Monograph 139, DHHS Pub No 94-3631.

[B37] MacKinnon DP, Fairchild AJ, Fritz MS (2007). Mediation Analysis. Annu Rev Psychol.

[B38] MacKinnon DP, Rose JS, Chassin L, Presson CC, Sherman SJ (2000). Contrasts in multiple mediator models. Multivariate applications in substance use research: new methods for new questions.

[B39] Lewis BA, Marcus BH, Pate RR, Dunn AL (2002). Psychosocial mediators of physical activity behavior among adults and children. Am J Prev Med.

[B40] Cheung C, Wyman J, Gross C, Peters J, Findorff M, Stock H (2007). Exercise behavior in older adults: a test of the transtheoretical model. J Aging Phys Act.

[B41] Elavsky S, McAuley E, Motl RW, Konopack JF, Marquez DX, Hu L, Jerome GJ, Diener E (2005). Physical activity enhances long-term quality of life in older adults: efficacy, esteem, and affective influences. Ann Behav Med.

[B42] McAuley E, Konopack JF, Morris KS, Motl RW, Hu L, Doerksen SE, Rosengren K (2006). Physical activity and functional limitations in older women: influence of self-efficacy. J Gerontol B Psychol Sci Soc Sci.

[B43] Resnick B, Orwig D, Hawkes W, Shardell M, Golden J, Werner M, Zimmerman S, Magaziner J (2007). The relationship between psychosocial state and exercise behavior of older women 2 months after hip fracture. Rehabil Nurs.

[B44] Umstattd MR, Hallam J (2007). Older adults' exercise behavior: roles of selected constructs of social-cognitive theory. J Aging Phys Act.

[B45] Balfour JL, Kaplan GA (2002). Neighborhood environment and loss of physical function in older adults: evidence from the Alameda County Study. Am J Epidemiol.

[B46] Bowling A, Barber J, Morris R, Ebrahim S (2006). Do perceptions of neighbourhood environment influence health? Baseline findings from a British survey of aging. J Epidemiol Community Health.

[B47] Wilcox S, Castro C, King AC, Housemann R, Brownson RC (2000). Determinants of leisure time physical activity in rural compared with urban older and ethnically diverse women in the United States. J Epidemiol Community Health.

[B48] King AC, Castro C, Wilcox S, Eyler AA, Sallis JF, Brownson RC (2000). Personal and environmental factors associated with physical inactivity among different racial-ethnic groups of U.S. middle-aged and older-aged women. Health Psychol.

[B49] Lochner K, Kawachi I, Kennedy BP (1999). Social capital: A guide to its measurement. Health Place.

[B50] Kawachi I, Kennedy BP, Lochner K, Prothow-Stith D (1997). Social capital, income inequality, and mortality. Am J Public Health.

[B51] Kawachi I, Kennedy B, Glass R (1999). Social capital and self-rated health: A contextual analysis. Am J Public Health.

[B52] Sundquist J, Johansson S, Yang M, Sundquist K (2006). Low linking social capital as a predictor of coronary heart disease in Sweden: A cohort study of 2.8 million people. Soc Sci Med.

[B53] Corburn J (2004). Confronting the challenges in reconnecting urban planning and public health. Am J Public Health.

[B54] Skjaeveland O, Garling T (1997). Effects of interactional space on neighbouring. J Environ Psychol.

[B55] Macintyre S, Ellaway A (2000). Neighbourhood cohesion and health in socially contrasting neighbourhoods: implications for the social exclusion and public health agendas. Health Bull (Edinb).

[B56] King WC, Belle SH, Brach JS, Simkin-Silverman LR, Soska T, Kriska AM (2005). Objective measures of neighborhood environment and physical activity in older women. Am J Prev Med.

[B57] Lim K, Taylor L (2005). Factors associated with physical activity among older people: a population-based study. Prev Med.

[B58] Michael YL, Beard TE, Choi D, Farquhar SA, Carlson NE (2006). Measuring the influence of neighborhood environment on walking among older adults. J Aging Phys Act.

[B59] Tudor-Locke C, Williams JE, Reis JP, Pluto D (2002). Utility of pedometers for assessing physical activity: convergent validity. Sports Med.

